# Statins for the Primary Prevention of Anthracycline Cardiotoxicity: A Comprehensive Review

**DOI:** 10.1007/s11912-024-01579-6

**Published:** 2024-07-13

**Authors:** Varun Bhasin, Azin Vakilpour, Marielle Scherrer-Crosbie

**Affiliations:** Division of Cardiovascular Medicine and Thalheimer Center for Cardio-Oncology, Perelman Center for Advanced Medicine and Hospital of the University of Pennsylvania, 3400 Civic Center Boulevard, Philadelphia, PA USA

**Keywords:** Statins, Anthracyclines, Cardiotoxicity, Cardiomyopathy, Ejection Fraction

## Abstract

**Purpose of Review:**

The aim of this review is two-fold: (1) To examine the mechanisms by which statins may protect from anthracycline-induced cardiotoxicity and (2) To provide a comprehensive overview of the existing clinical literature investigating the role of statins for the primary prevention of anthracycline-induced cardiotoxicity.

**Recent Findings:**

The underlying cardioprotective mechanisms associated with statins have not been fully elucidated. Key mechanisms related to the inhibition of Ras homologous (Rho) GTPases have been proposed.

Data from observational studies has supported the beneficial role of statins for the primary prevention of anthracycline-induced cardiotoxicity. Recently, several randomized controlled trials investigating the role of statins for the primary prevention of anthracycline-induced cardiotoxicity have produced contrasting results.

**Summary:**

Statins have been associated with a lower risk of cardiac dysfunction in cancer patients receiving anthracyclines. Further investigation with larger randomized control trials and longer follow-up periods are needed to better evaluate the long-term role of statin therapy and identify the subgroups who benefit most from statin therapy.

## Introduction

Anthracyclines are an essential therapy for several types of cancer including breast cancer, lymphoma, leukemia, and sarcoma. As advances in cancer therapy have improved outcomes and survival in cancer patients, cancer therapy-related cardiac dysfunction (CTRCD) has become more apparent, occurring occurring in 5–30% of patients depending on the treatment, the population, and the definition of CTRCD [1, 2]. Anthracycline-induced cardiotoxicity (AIC) in particular has been associated with poor outcomes, including increased morbidity and mortality [2–4]. As a result, there is a significant need to identify cardioprotective agents that can help prevent or mitigate anthracycline-induced cardiotoxicity (AIC) [5].

Several studies have examined the use of heart failure treatments for the prevention of anthracycline-induced cardiomyopathy. These studies in anthracycline-treated patients have reported either a small or no significant effect on left ventricular ejection fraction (LVEF) [6–10]. The hemodynamic impact of neurohormonal therapies including angiotensin-converting enzyme inhibitors, angiotensin receptor blockers, and beta-blockers can make it challenging to prescribe these drugs in cancer patients undergoing chemotherapy. While dexrazoxane has been shown to be an effective cardioprotective agent [11], it has dose-limiting myelotoxicity concerns [12], and an increased risk of secondary cancer has also been reported [13]. There is mechanistic plausibility supporting the promising role of statins for the primary prevention of AIC, bolstered by experimental data [14–16].

Several observational studies and recent randomized controlled trials have investigated the impact of statins for the prevention of AIC. The objective of this review is two-fold: (1) To examine the potential mechanisms of AIC and cardioprotective mechanisms associated with statins and (2) To provide a comprehensive overview of the existing clinical literature investigating the role of statins for the primary prevention of anthracycline-induced cardiotoxicity. Additionally, this review also discusses challenges and limitations present in the current literature while providing insights into areas requiring further investigation.

## Potential Mechanisms of Anthracycline-Induced Cardiomyopathy

**Fig. 1:**
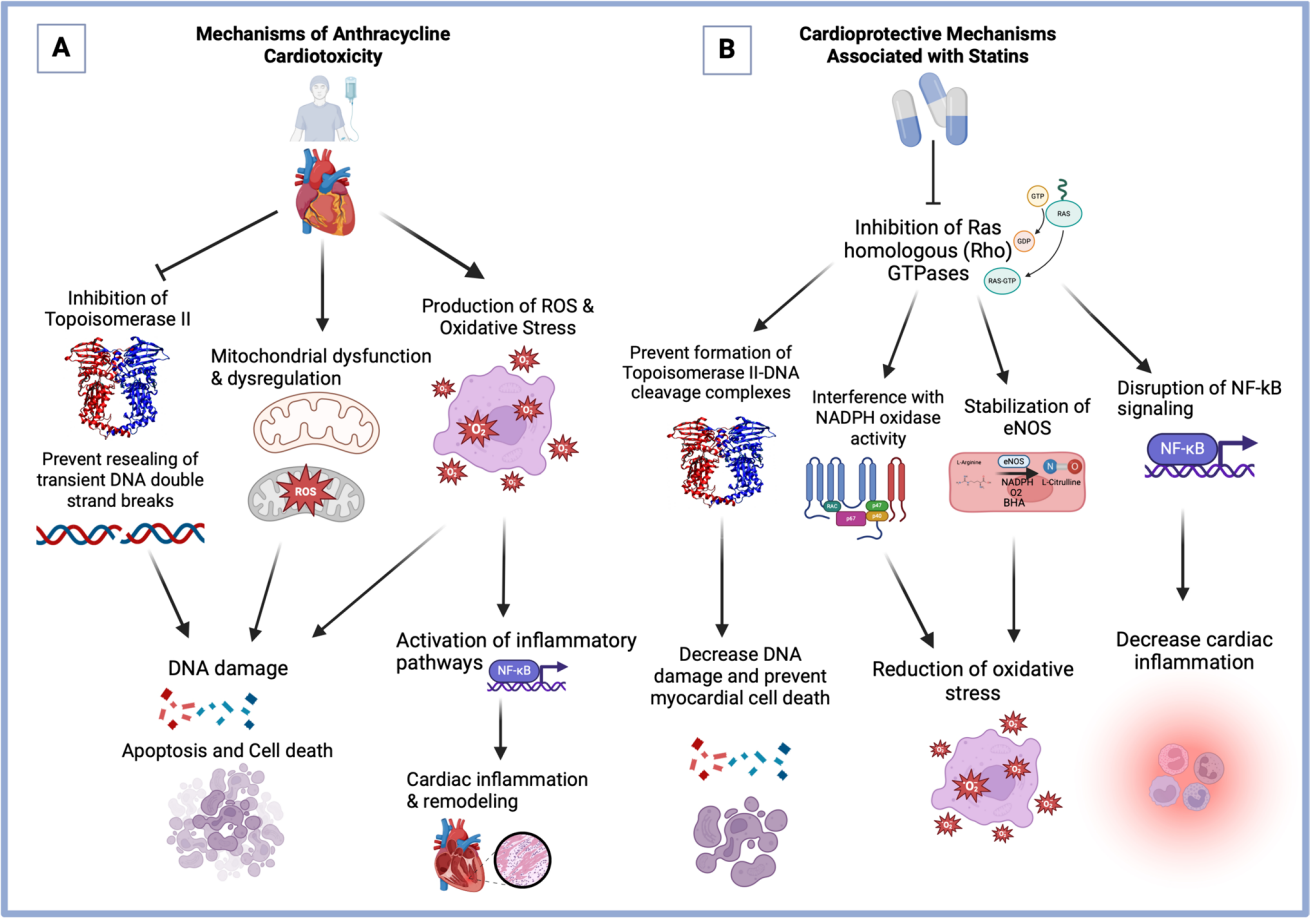
**A** Mechanisms of Anthracycline-Induced Cardiotoxicity. **B** Cardioprotective Mechanisms Associated with Statin Therapy

The mechanisms underlying anthracycline-induced cardiotoxicity have not been fully elucidated; however, several contributors have been proposed [15, 17] (Fig. 1A). The current literature supports the hypothesis that the pathophysiology of anthracycline-induced cardiomyopathy is largely dependent on the targeting of Topoisomerase II beta (TOP2B) in cardiomyocytes, an increase in oxidative stress, and the disruption of mitochondrial homeostasis [18, 19].

### Topoisomerase II Inhibition

TOP 2B has been suggested as the key mediator of AIC [17]. TOP2B cuts DNA double-strands allowing strand passage for unwinding and unknotting of supercoiled DNA to generate transient double-strand breaks. These cuts are usually resealed promptly following strand passage [20]. Anthracyclines bind and stabilize TOP2 DNA cleavage complexes, preventing the resealing of transient DNA strand breaks. This can stimulate an apoptotic response and trigger cardiomyocyte death [18, 20, 21]. Experimental studies have shown that mice with TOP2B knockout experienced less cardiotoxicity, no decline in LVEF and reduced mitochondrial dysfunction following doxorubicin therapy [18, 22].

### Oxidative Injury/Free Radical Generation and DNA Damage

One of the most accepted hypotheses explaining anthracycline-induced cardiotoxicity is the production of free radicals and ROS via several mechanisms [17, 23]. Doxorubicin can induce the production of free radicals and activation of ROS by redox cycling that is catalyzed by enzymes including NADPH oxidase (NOX) and mitochondrial NADH dehydrogenase, ultimately leading to DNA damage and apoptosis [24–26]. Additionally, increased levels of NO produced by isoforms of endothelial nitric oxide synthase (eNOS) and inducible nitric oxide synthase (iNOS) have been found in cardiac cells during doxorubicin therapy [27]. Superoxide anion generated by activated NOX reacts with the NO leading to the formation of peroxy-nitrite oxides which further contribute to oxidative stress, cell necrosis, and apoptosis [27]. Anthracyclines can form iron-anthracycline complexes, which further catalyze the production of various ROS [18]. Moreover, doxorubicin can enhance inflammatory mediators via activation of NFkB through oxidative stress. This can result in cardiac remodeling and cardiomyopathy [24, 27].

### Disruption of Mitochondrial Function

Anthracyclines can disrupt mitochondrial function by uncoupling the electron transport chain, affecting the mitochondrial membrane potential, and increasing the production of reactive oxygen species (ROS) [28–30]. Long-lasting mitochondrial homeostasis dysfunction may also add to pathophysiology of chronic and late-onset anthracycline induced heart failure [18]. Additionally, doxorubicin has been shown to interfere with mitochondrial metabolism and gene expression [28–30].

## Potential Cardioprotective Mechanisms Associated with Statins

Statins have the potential to prevent cardiovascular toxicity independent of their LDL-cholesterol lowering effects (pleiotropic effects) (Fig. 1B). Statins can prevent anthracycline-induced cardiotoxicity through mechanisms involving antioxidant, anti-inflammatory, and geno-protective effects [18, 31].

One of the most important mechanisms of cardio protection is the inhibition of specific proteins called Ras homologous (Rho) GTPases [18, 31, 32]. Rho GTPases act as molecular switches and play a key role in the regulation of several cellular processes [33]. Inhibition of Rho GTPase signaling, particularly Rac1 inhibition, can decrease the activity of NADPH oxidase complex and stabilize eNOS, thereby reducing ROS production and decreasing inflammation [18, 34]. In addition, statins can stimulate the expression of genes coding for antioxidant factors like superoxide dismutase 2 and subsequently reduce oxidative stress [18, 35]. Statins can also prevent DNA damage and myocardial cell death via the inhibition of Rho GTPases. The inhibition of Rac1 prevents the formation of topoisomerase II-DNA cleavage complexes, consequently decreasing DNA damage and apoptosis [36, 37]. Regarding the anti-inflammatory mechanism of statins, in vitro studies have demonstrated that statins inhibit the nuclear translocation of the proinflammatory transcription factor NF-kB, a process mediated through the inhibition of Rho GTPases [18, 38]. Thus, statins can mitigate anthracycline-induced cardiac inflammation and cell death by disrupting NF-kB signaling, interfering with NADPH oxidase and eNOS activity, and upregulating the DNA damage response [18].

## Observational Studies

Several retrospective studies supported the role of statins for the primary prevention of AIC (Table 1). Three propensity matched cohort studies, totaling 762 patients, demonstrated a lower risk of incident heart failure in patients receiving anthracyclines and continuous statin therapies [39–41]. Two of these studies were conducted in breast cancer patients [39, 40]. Another study revealed a lower rate of heart failure in both gastric and breast cancer patients receiving statin therapy [41]. Furthermore, a prospective cohort study of patients with breast, lymphoma, and leukemia patients treated with anthracyclines concluded that there was no significant change in LVEF in patients concomitantly treated with a statin for clinical reasons and a -6.5% ± 1.5% decline in LVEF in the cohort that did not receive a statin [42]. As result, the favorable findings of these small observational studies set the stage for randomized trials investigating the cardioprotective role of statins in cancer patients receiving anthracyclines.

**Table 1:** Observational Studies Evaluating Impact of Statins on Cardiotoxicity in Patients Receiving Anthracycline Therapy

Author (year)	Type	Malignancy	Number of Patients (N)	Age (years) Mean ± SD	Statin	Imaging Modality	Endpoint	Duration	Results
Seicean (2012) [39]	Propensity matched cohort	Breast	201	60 ± 9	All	–-	HF hospitalization	2.55 ± 1.7 years	HR = 0.3 (95% CI, 0.1–0.9; p = 0.03)
Abdel-Qadir (2020) [40]	Propensity matched cohort	Breast	129	69 (median age)	All	–-	HF hospitalization	5.1 ± 3.1 years	HR = 0.45 (95% CI, 0.24–0.85; *p* = 0.01)
Tase (2013) [41]	Propensity matched cohort	Breast Gastric	432	53.5 ± 1	Atorvastatin Rosuvastatin Other	–-	HF hospitalization	2.55 ± 1.7 years	Rate of HF 4.9% (statin) vs. 9.0% (placebo), dispersion 0.05, and 0.08
Chotenimitkhun (2015) [42]	Prospective cohort	Breast Leukemia Lymphoma	51	48 ± 2	AtorvastatinSimvastatin	cMRI	LVEF	6 months	Change LVEF 1.1 ± 2.6% (statin) vs -6.5 ± 1.5% (placebo), *p* = 0.03

*HF* Heart failure, *cMRI* cardiac magnetic resonance imaging, *LVEF* Left ventricular ejection fraction, *HR* Hazard ratio

## Randomized Controlled Trials

In recent years, several randomized control trials (RCTs) investigating the impact of statins on the development of anthracycline induced cardiotoxicity have been conducted. In 2011, a small un-blinded RCT was completed by Acar et al. [43]. The next RCT was then completed by Nabati et al. in 2019 [44]. Following these early trials, four additional randomized trials were completed during the last two years. These trials include the Preventing Anthracycline Cardiovascular Toxicity with Statins (PREVENT) trial in 2022, Statins to Prevent the Cardiotoxicity of Anthracyclines (STOP-CA) in 2023, Statins for the Primary Prevention of Heart Failure in Patients Receiving Anthracycline-based Chemotherapy (SPARE-HF) in 2023, and a RCT by Mohamed et al. in 2023 (Table 2) [45–48]. These RCTs examining the impact of statins on the development of anthracycline-induced cardiotoxicity produced contrasting results. While Acar et al., Nabati et al., the STOP-CA trial, and Mohamed et al. demonstrated a beneficial role for statin therapy, the PREVENT and SPARE-HF trials did not show any significant benefit with statin therapy.

**Table 2:** Randomized Controlled Trials Evaluating Impact of Statins on Cardiotoxicity in Patients Receiving Anthracycline Therapy

Author (year)	Type	Malignancy	Number of Patients (N)	Age (years) Mean ± SD	Statin, dose (mg)	Imaging Modalities	Endpoint	Duration	Results
Acar et al. (2011) [43]	Un-blinded RCT	Non-Hodgkin Lymphoma (57.5%) Multiple Myeloma (15%) Leukemia (27.5%)	40	53 ± 15	Atorvastatin 40 mg	Echo	LVEF	6 months	Change in LVEF: 1.3 ± 3.8% (statin) vs. -7.9 ± 8.0% (control) p < 0.001
Mohamed et al. (2023) [48]	Single- blinded RCT	Breast (100%)	110	53 ± 15	Atorvastatin 40 mg	Echo	LVEF	6 months	Change in LVEF: 3.6% (statin) vs. -7.1% (placebo) *p* = 0.004
Nabati et al. (2019) [44]	Single- blinded RCT	Breast (100%)	89	49.3 ± 11.2	Rosuvastatin 20 mg	Echo	LVEF	6 months	Change in LVEF: -1.5 ± 6.6% (statin) vs. -5.2 ± 5.7% (placebo) *p* = 0.012
PREVENT (2022) [45]	Double-blinded RCT	Breast (85%) Lymphoma (15%)	279	49 ± 12	Atorvastatin 40 mg	cMRI	LVEF	24 months	No significant difference in LVEF decline: -3.2 ± 0.7% (statin) vs. -3.3 ± 0.6% (placebo) *p* = 0.93
STOP-CA (2023) [46]	Double- blinded RCT	Lymphoma (100%)	300	50 ± 17	Atorvastatin 40 mg	cMRI (primary modality) Echo	LVEF	12 months	Proportion of patients with LVEF decrease ≥ 10% to EF < 55%: 9% (statin) vs. 22% (placebo) *p* = 0.002
SPARE-HF (2023) [47]	Double-blinded RCT	Breast (65%) Lymphoma (21%) Sarcoma (6%) Thymoma (5%) Leukemia (3%)	112	56.9 ± 13.6	Atorvastatin 40 mg	cMRI	LVEF	2.5 months	No significant difference in LVEF: 57.3 ± 5.8% (statin) vs. 55.9 ± 7.4% (placebo) *p* = 0.34

*cMRI* cardiac MRI, *RCT* Randomized controlled trial, *HF* Heart failure, *LVEF* Left ventricular ejection fraction

A small initial trial by Acar et al. completed in 2011 found that atorvastatin could be effective in preventing the development of cardiomyopathy in patients treated with anthracyclines. 40 patients with either non-Hodgkin’s lymphoma, multiple myeloma, or leukemia were randomized into a statin group or control group (non-placebo). Patients in the statin group received 40 mg of Atorvastatin prior to chemotherapy and for a total of 6 months. The primary endpoint was defined as the development of a cardiomyopathy (EF < 50%) as identified by 2D echocardiography. The mean change in LVEF in the statin group after 6 months was 1.3 ± 3.8% versus -7.9 ± 8.0% in the control group (*p* < 0.001). This study was limited by its small sample size, heterogeneous patient population, and lack of a placebo group [43].

A subsequent randomized, single-blinded, placebo-controlled trial by Nabati et al. in 2019 randomized 89 females with newly diagnosed breast cancer receiving anthracycline chemotherapy. Patients received 20 mg of rosuvastatin 24 h prior to the first cycle of chemotherapy and once daily during the follow-up period. The primary endpoints were change in LVEF and global longitudinal strain (GLS) on echocardiography after completion of chemotherapy when compared with baseline values. There was a significant reduction in LVEF in the placebo group, however, there was no significant change in the LVEF compared to baseline in the statin group (inter-group *p* = 0.012). There was no significant difference in GLS between the groups after chemotherapy [44]. Overall, this trial supported that prophylactic use of rosuvastatin in patients with breast cancer may prevent chemotherapy-induced cardiotoxicity.

The Statins to Prevent the Cardiotoxicity of Anthracyclines (STOP-CA) trial recently demonstrated a lower rate of cardiac systolic dysfunction in patients with lymphoma receiving anthracyclines and prophylactic atorvastatin. This multi-center double-blind, placebo-controlled randomized trial enrolled 300 patients with lymphoma being treated with anthracyclines with a median dose of 300 mg/m^2^. Patients in the statin group received atorvastatin 40 mg daily prior to the initiation of anthracycline treatment and continued statin therapy for 12 months. The primary endpoint was the proportion of patients who experienced an absolute decline in LVEF of ≥ 10% (primarily based on cardiac MRI) from baseline to a final value of < 55% at 12 months. A secondary outcome was the proportion of patients with an absolute decline in LVEF of ≥ 5% from baseline to a final value of < 55% over 12 months. Patients who received atorvastatin had a significantly lower incidence of cardiac dysfunction (defined as the primary endpoint) compared to those who received placebo (9% versus 22%, *p* = 0.002). The odds of reaching the primary endpoint were almost 3 times greater for patients randomized to the placebo group compared to those who were in the atorvastatin group (OR, 2.9, 95% CI 1.4 – 6.4) [46]. This trial also demonstrated that older age, female gender, and higher anthracycline dose were predictive of the beneficial impact of atorvastatin on post-treatment LVEF. However, the STOP-CA trial did not enroll a racially or ethnically diverse population [46].

A recent single-blinded RCT utilizing 3D echocardiography by Mohamed et al. enrolled 110 female patients with newly diagnosed breast cancer who received anthracycline based chemotherapy at a single center in Egypt. Patients were randomized to 40 mg of atorvastatin or placebo. Comprehensive transthoracic echocardiography was performed at baseline and after 6 months of anthracycline chemotherapy, and 3D echocardiography was used to assess ejection fraction. CTRCD was defined as a drop in LVEF of ≥ 10% to a value < 53% based on 3D echo. Only 12% of patients developed CTRCD in the statin group while 30% of patients in the placebo/control group developed CTRCD. This study concluded that atorvastatin may prevent the development of CTRCD in patients with breast cancer receiving anthracyclines. Limitations of this study include its small sample size, single-blinded study design, and lack of multi-center enrollment [48].

The PREVENT trial investigated the efficacy of atorvastatin in 279 patients with breast cancer or lymphoma receiving doxorubicin. Most patients in the PREVENT trial participants had breast cancer (85%). 90% of the patients in this trial were treated with anthracyclines at a median cumulative dose of 243 mg/m^2^. The primary endpoint was the absolute LVEF decrease at 24 months, as measured by cardiac MRI. There was no significant difference in the decline in LVEF between the statin and the placebo groups [45].

Finally, SPARE-HF was a smaller, multi-center RCT which enrolled 112 patients with different cancers (including breast, lymphoma, sarcoma, thymoma, and leukemia) who were at increased risk for CTRCD based on the American Society of Clinical Oncology (ASCO) clinical guidelines [47]. A median anthracycline dose of 243 mg/m^2^ was administered. The primary endpoint was LVEF (measured using cMRI) after completion of anthracycline therapy in both groups. There was no significant difference in LVEF between the atorvastatin and placebo groups after adjusting for the baseline LVEF (0.79% difference, *p* = 0.34). Of note, the SPARE-HF trial had a median follow-up period of only 72 days [47].

Several reasons may explain a difference between the PREVENT and the STOP-CA trials. In the STOP-CA trial, patients received a median anthracycline dose of 300 mg/m^2^. In contrast, patients in the PREVENT trial (and the SPARE-HF trial) received lower median anthracycline doses of 240 mg/m^2^ and 243 mg/m^2^, respectively [45, 47]. Anthracycline-induced cardiotoxicity occurs in a dose dependent manner, with an increased incidence of cardiotoxicity at cumulative doses > 250 mg/m^2^. As a result, cardiotoxicity is higher and the beneficial effect of statins may be more easily detectable in patients exposed to higher cumulative dose of anthracyclines. The choice of a binary endpoint (presence of significant cardiac dysfunction as opposed to the decrease of LVEF as a continuous variable) may have helped to identify a population at risk and prevent dilution of the statin effect. Finally, the PREVENT trial experienced a significant drop-out rate and decrease in therapeutic adherence that may have influenced the outcome [1, 45, 46].

## Meta-Analyses

Two recent meta-analyses have confirmed an overall beneficial effect of statins in anthracycline-induced cardiotoxicity. One meta-analysis of both observational studies and RCTs demonstrated a reduced incidence of cardiotoxicity as a binary endpoint in patients receiving anthracyclines and treated with statins [49]. Another meta-analysis examined recent randomized controlled trials and demonstrated a beneficial impact of statins on the risk of anthracycline-induced cardiomyopathy and LVEF preservation [1].

## Statin Choice and Dose

High-intensity statin regimens were utilized in all the recent RCTs [43–48]. Atorvastatin 40 mg was used in all RCTs, except for the trial by Nabati et al. which employed rosuvastatin 20 mg [44]. In a recent meta-analysis of RCTs, a leave-one sensitivity analysis with removal of the trial by Nabati et al. did not negatively impact the results for the efficacy endpoints [1]. This suggests that both high intensity atorvastatin and rosuvastatin may be effective in preventing CTRCD. Other statins or lower-intensity statin regimens have not been evaluated in RCTs.

## Clinical Implications

The current ESC guidelines recommend the use of statins for primary prevention in patients treated with anthracyclines who are at high and very-high risk for cardiovascular toxicity (class IIA recommendation, level of evidence B) [1]. The results of the recent STOP-CA trial suggest that high-risk patients, including those who are older, those with a borderline baseline LVEF, and those receiving higher doses of anthracyclines, are most likely to benefit from statin therapy [46].

## Conclusions

In conclusion, statins have been associated with a lower risk of cardiac dysfunction in cancer patients receiving anthracyclines. Recent RCTs have produced promising results, but additional RCTs are needed. Larger studies are necessary to understand which groups would benefit most from statins for the prevention of anthracycline-induced cardiac dysfunction and whether the beneficial effect is sustained. Further studies should also examine whether statins have synergistic effects with other cardioprotective agents. Finally, for widespread acceptance, it will be crucial to confirm that the beneficial impact of statins on cardiac function translates into a decrease in morbidity or mortality in patients with cancer treated with anthracyclines.
